# Accuracy mechanism of eukaryotic ribosome translocation

**DOI:** 10.1038/s41586-021-04131-9

**Published:** 2021-12-01

**Authors:** Muminjon Djumagulov, Natalia Demeshkina, Lasse Jenner, Alexey Rozov, Marat Yusupov, Gulnara Yusupova

**Affiliations:** 1 Institute of Genetics and Molecular and Cellular Biology, CNRS UMR7104, INSERM U1258, University of Strasbourg, Illkirch, Strasbourg France; 2 Urania Therapeutics, Ostwald, France; 3 Present Address: Biochemistry and Biophysics Center, National Heart, Lung and Blood Institute, Bethesda, MD USA

**Keywords:** Ribosome, X-ray crystallography

## Abstract

Translation of the genetic code into proteins is realized through repetitions of synchronous translocation of messenger RNA (mRNA) and transfer RNAs (tRNA) through the ribosome. In eukaryotes translocation is ensured by elongation factor 2 (eEF2), which catalyses the process and actively contributes to its accuracy^1^. Although numerous studies point to critical roles for both the conserved eukaryotic posttranslational modification diphthamide in eEF2 and tRNA modifications in supporting the accuracy of translocation, detailed molecular mechanisms describing their specific functions are poorly understood. Here we report a high-resolution X-ray structure of the eukaryotic 80S ribosome in a translocation-intermediate state containing mRNA, naturally modified eEF2 and tRNAs. The crystal structure reveals a network of stabilization of codon–anticodon interactions involving diphthamide^1^ and the hypermodified nucleoside wybutosine at position 37 of phenylalanine tRNA, which is also known to enhance translation accuracy^2^. The model demonstrates how the decoding centre releases a codon–anticodon duplex, allowing its movement on the ribosome, and emphasizes the function of eEF2 as a ‘pawl’ defining the directionality of translocation^3^. This model suggests how eukaryote-specific elements of the 80S ribosome, eEF2 and tRNAs undergo large-scale molecular reorganizations to ensure maintenance of the mRNA reading frame during the complex process of translocation.

## Abstract (web summary)

Structural analysis of the *Saccharomyces cerevisiae* 80S ribosome trapped in an intermediate translocation state shows stabilization of codon–anticodon interactions by eukaryote-specific elements of the 80S ribosome, eEF2 and tRNA and demonstrates a major role for eEF2 in maintaining the directionality of translocation.

## Main

In eukaryotes, the complex process of translocation is ensured by eEF2, a GTPase that is indispensable for maintaining the correct mRNA reading frame. Many genetic and biochemical studies point to a critical role of the unique eEF2 post-translational modification diphthamide, which is located in domain IV and is conserved among eukaryotes and archaea. Organisms lacking diphthamide have reduced protein synthesis rates and increased occurrence of (−1) frameshifting^1,4^. Diphthamide is a target of several virulent toxins that inactivate eEF2 by ADP ribosylation and cause lethal effects^5^.

The current structural knowledge about dynamics of the eukaryotic elongation cycle has been provided by low-to-intermediate resolution cryo-electron microscopy (cryo-EM) reconstructions^6–10^. Although several late steps of translocation have been described, the level of detail achieved in these studies is insufficient to suggest a precise mechanism explaining fidelity of translocation. At the same time, understanding of the principles of the fidelity has become particularly crucial during the current viral pandemic, because programmed mRNA frameshifting is at the heart of the SARS-CoV-2 replication cycle^11^.

Here we present the crystal structure of eukaryotic 80S ribosomes from *Saccharomyces cerevisiae* trapped in intermediate translocation state. The X-ray crystal structure provides a detailed mechanism of tRNA translocation from A- to P-sites and highlights the specific role of eEF2 in the movement of the tRNA–mRNA module during the process. The crystal structure presented here uncovers the precise role of diphthamide and wybutosine, a heavily modified nucleoside at position 37 of eukaryotic phenylalanine tRNA, in stabilization of the codon–anticodon interactions during translocation and demonstrates how eukaryote-specific elements of the 80S ribosome, eEF2 and tRNA rearrange to ensure maintenance of the mRNA reading frame.

## Architecture of the translocation complex

**Fig. 1:**
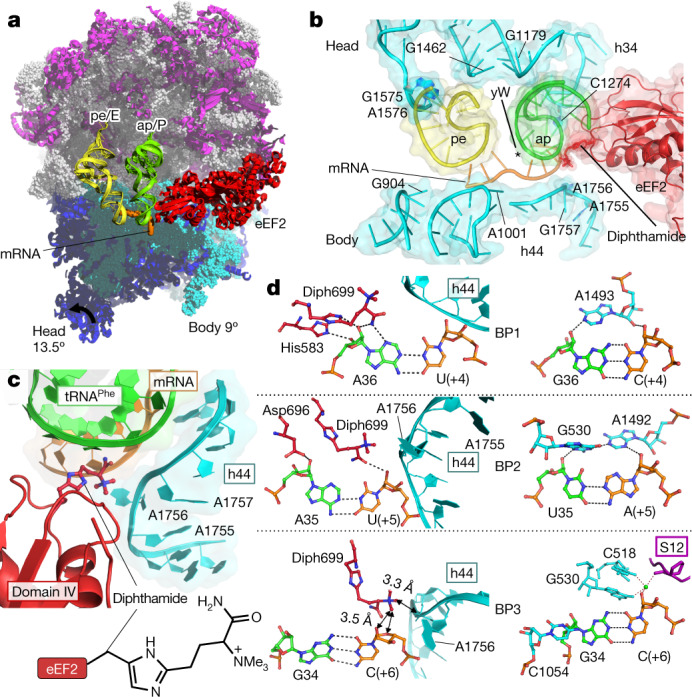
The translocation-intermediate state of the eukaryotic 80S ribosome with eEF2–GMPPCP, mRNA and tRNAs,, showing diphthamide of eEF2 is involved in stabilizing codon–anticodon interactions early in translocation. **a**, Overview of the translocation-intermediate complex with two tRNAs trapped in chimeric hybrid ap/P and pe/E states. **b**, Close-up view of ap/P and pe/E tRNA anticodon stem loops in the context of elements of the SSU body and head and domain IV of eEF2. Position of wybutosine (yW) of ap/P tRNA^Phe^ is indicated by the asterisk. **c**, Position of diphthamide (Diph699) at the conjunction of the AAG anticodon of ap/P tRNA^Phe^, mRNA codon UUC and decoding adenosines 1755–1756 of helix 44 (h44). **d**, Stabilization networks around codon–anticodon interactions in the translocation-intermediate complex (left) and the decoding centre of the bacterial ribosome in the cognate classical state^13,14^ (right). Top, middle and bottom panels depict stabilization around the first, second and third base pairs (BP1–3) of a codon–anticodon duplex, respectively. Conserved adenosines 1755 and 1756 in yeast 18S rRNA correspond to adenosines 1492 and 1493 in 16S rRNA of the bacterial decoding centre. eEF2 is shown in red, mRNA is in orange, chimeric hybrid ap/P tRNA is in green and chimeric hybrid pe/E is in yellow. LSU rRNA and proteins are shown in grey and purple; SSU rRNA and proteins are in cyan and in deep blue. Degrees of SSU body and head rotations are indicated and were obtained by superimposing with the non-rotated 80S ribosome (PDB ID 3J78).

**Extended Data Fig. 1:**
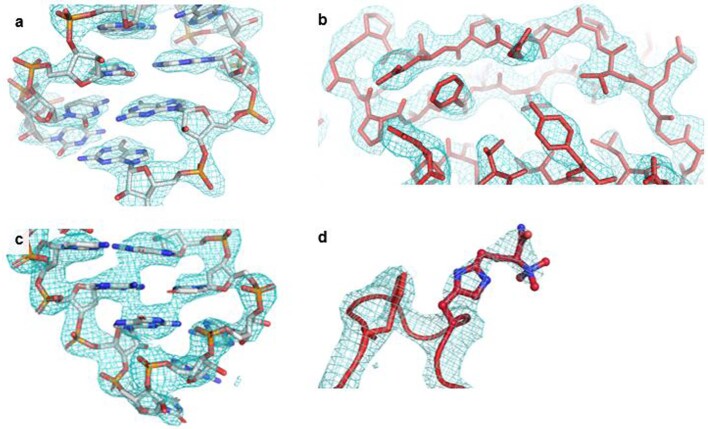
Examples of the electron density maps of the 80S ribosome translocation intermediate complex. FEM^36^ map for a representative part of 25S rRNA (**a**) and for eEF2 (**b**) contoured at 2 sigma. *2F*_*o*_*-F*_*c*_ electron density map contoured at 1.5 sigma for 25S rRNA (**c**) and at 1 sigma for the diphthamide loop of eEF2 (**d**).

We determined the structure of the *S. cerevisiae* 80S ribosome translocation complex trapped in intermediate state by X-ray crystallography at 3.2 Å resolution (Fig. 1a, b, Extended Data Fig. 1, Extended Data Table 1). It consists of *S. cerevisiae* 80S ribosomes bound with native *S. cerevisiae* eEF2, the nonhydrolyzable GTP analogue GMPPCP, mRNA and two tRNAs, and was determined in the absence of antibiotics, which customarily used for stabilization, suggesting that the model represents a bona fide state of the 80S ribosome.

**Extended Data Fig. 2:**
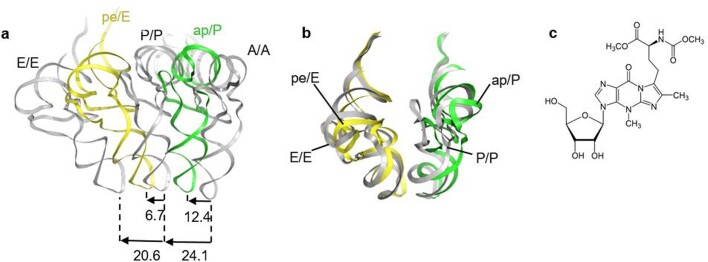
Comparison of various states of tRNAs on the ribosome. **a**, Positions of tRNAs of the intermediate translocation complex relative to tRNAs in the classical A, P and E states (grey, PDB ID 4V6F) aligned on the SSU body; indicated distances are in Å. **b**, Positions of tRNAs relative to tRNAs in the classical nonrotated state (PDB ID 3J78) aligned on LSU. **c**, Chemical structure of the wybutosine tRNA^Phe^ modification at position 37.

The crystal structure of the ribosome complex represents an intermediate translocation state that has not been described before with two tRNAs trapped in the chimeric hybrid ap/P and pe/E transitory positions. In this state the small subunit (SSU) head has swivelled 13.5° and the SSU body has undergone 9° anticlockwise rotation relative to the large subunit (LSU) (Fig. 1a). The anticodon stem–loop (ASL) of the A-site tRNA is captured half-translocated between the A- and P-sites of SSU (12.4 Å out of a fully translocated distance of 24.1 Å), and the tRNA acceptor end contacts the P-loop of the peptidyl-transferase centre of LSU forming an ap/P chimeric hybrid state (Fig. 1a, b, Extended Data Fig. 2a, b).

**Extended Data Fig. 3:**
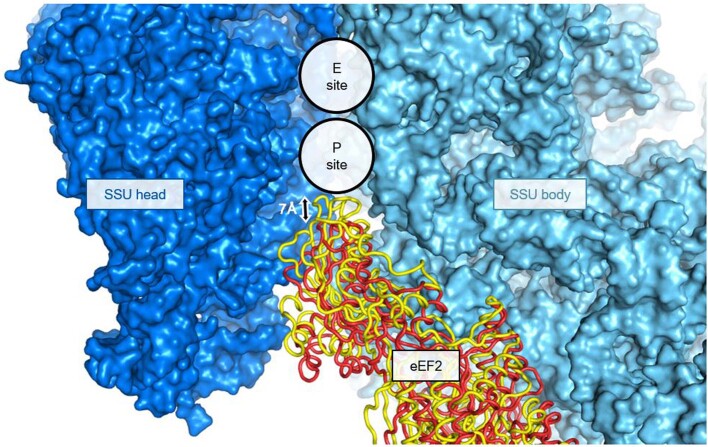
Insertion of eEF2 into the SSU A site in the late translocation complex TI-POST1 (yellow, PDB ID 6GZ3) in comparison to the position of eEF2 in the early translocation intermediate state that we determined in this study (red). View from the LSU side. Alignment is performed using the SSU body.

**Extended Data Fig. 4:**
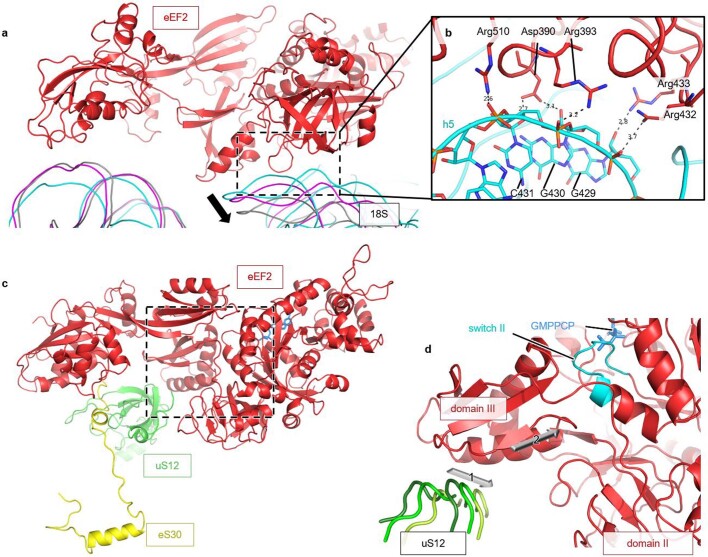
Interactions of eEF2 with the SSU body. **a**, Comparison of the current X-ray structure of an early translocation intermediate (18S rRNA in cyan) with cryo-EM models of late translocation steps TI-POST1 (magenta, PDB ID 6GZ3) and TI-POST3 (grey, PDB ID 6GZ3)^6^. Superimposition of eEF2 in the two structures shows that there is a noticeable shift of 18S rRNA of the SSU body away from eEF2 at later stages of translocation. **b**, Contacts between eEF2 and h5 of SSU of the rotated ribosome in the reported intermediate translocation complex. **c**, Interactions between eEF2 and the SSU shoulder proteins eS30 and uS12. Stabilization of the eEF2 domain IV by the N-terminus of eukaryote-specific protein eS30 that itself interacts with conserved decoding protein uS12. Presumably, eS30 co-evolved with eEF2, whose domain IV has 65 additional amino acids compared to its bacterial counterpart EF-G^5^, to provide supplementary stabilization as well as to enhance propagation of conformational changes at the decoding site^13,14^. **d**, A close up view of the dashed region from (**c**). Movement of uS12 (arrow 1) induced by the SSU body back-rotation can propagate to switch II (in cyan) of eEF2 through domain III (arrow 2) and can trigger GTP hydrolysis or Pi release. Regions of uS12 adjacent to eEF2 are shown and coloured dark- to light-green based on the rotational state where dark-green is the most rotated state (intermediate translocation complex) and medium (TI-POST1) and light (TI-POST3) greens represent the least rotated states. Alignment was done using eEF2.

The overall conformation of eEF2 resembles that of eEF2 in the 80S translocation intermediate–post-translocation (TI-POST)-1 state of a medium-resolution cryo-EM reconstruction^6^ that depicts a late-translocation event with domain IV of eEF2 protruding approximately 7 Å deeper into the A-site of SSU from the perspective of the SSU body (Extended Data Fig. 3). However—in contrast to the TI-POST-1 state—in our complex, domain IV of eEF2 is engaged in an extensive stabilization network with the codon–anticodon duplex and with the decoding centre area (Fig. 1b–d, Extended Data Fig. 1d). The intricate network of arginine-rich regions of domain II and III of eEF2 with helix 5 of 18S rRNA is formed owing to strong rotation of the SSU body (9.85°). These contacts are not present in the less rotated (4.26°) TI-POST-1 or unrotated TI-POST-3 states of 80S from the cryo-EM models^6^ (Extended Data Fig. 4a, b). This comparison suggests that eEF2 uncouples from the SSU body at the later steps of translocation where it is associated only with LSU and remains stationary relative to the rest of the ribosome. Collectively, these structural data corroborate an existing hypothesis describing a translocase (eEF2 or EF-G) as a ‘locking pawl’ that decouples tRNA–mRNA from the SSU head and body during back-rotation^3^.

**Extended Data Fig. 5:**
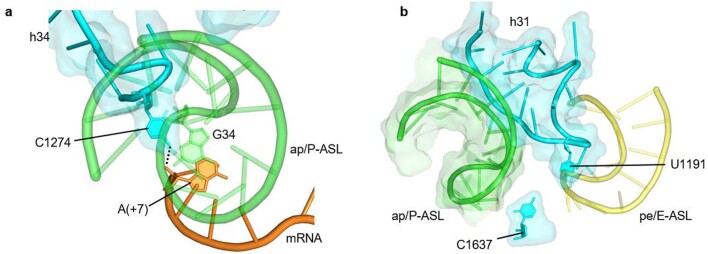
Rearrangement of h34 and h31 of SSU with ASL movement of tRNAs in the translocation intermediate complex. **a**, A new position of conserved C1274 in h34 of 18S rRNA (C1054 of 16S rRNA in bacteria, Fig. 1d) relative to ASL of the A site tRNA and mRNA. By establishing a contact with mRNA nucleotide in position (+7) C1274 can contribute to maintaining mRNA reading frame during translocation. **b**, In the early intermediate state of translocation h31 of 18S rRNA moves together with pe/E tRNA. In this state, the U1191-C1637 interaction is broken, however, U1191 remains in contact with C35 of pe/E tRNA. A similar situation was described for the bacterial ribosome with the pe/E tRNA in a structure modelling spontaneous translocation without EF-G^37^. Compared to the intermediate eukaryotic state reported here, the bacterial ap/P tRNA coupled to mRNA is shifted towards the P-site indicating translational reading frame slippage in the factor-free system. This comparison shows the importance of role that translocase eEF2 (or EF-G) fulfils in the coordinated movement of tRNA-mRNA during translocation.

**Extended Data Fig. 6:**
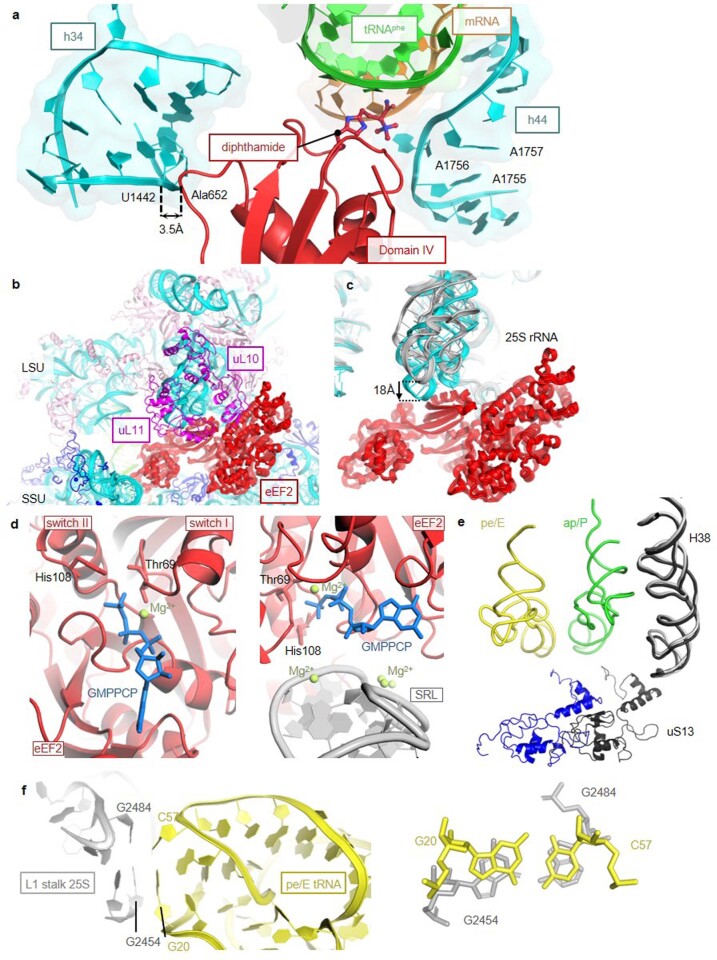
Contacts between eEF2, tRNAs and the 80S ribosome in the crystal structure of the translocation intermediate complex. **a**, Position of diphthamide at the conjunction of ap/P tRNA^Phe^, mRNA and decoding adenines of h44 and interactions of eEF2 (Ala652) with rRNA of the SSU head (U1442 of 18S). **b**, A solvent view on the P stalk region. **c**, Movement of the P stalk upon eEF2 binding seen when comparing our structure to that of the vacant 80S ribosome structure (PDB ID 4V88). Alignment was performed using 25S rRNA as a reference. **d**, The G domain of eEF2 with ordered switch I and II regions indicating that this is a pre-hydrolysis state (left panel). Close-up view of the GTP pocket and sarcin-ricin loop (SRL) of 25S rRNA (right panel). **e**, Disruption of the B1a bridge consisting of helix 38 of 25S rRNA (A-site finger) and protein uS13 induced by rotation of the SSU head and body. Non-rotated 80S (PDB ID 3J78) is coloured in black. **f**, Stacking interactions of rRNA elements (magnified in the right panel) of the L1 stalk with the elbow region of pe/E tRNA.

Comparison of the current early-intermediate state with late-translocation states described by cryo-EM^6^ reveals that the uS12 protein remains attached to domain III of eEF2 during SSU back-ratcheting. Located on the SSU shoulder, uS12 is implicated in translocation^12^ and codon–anticodon duplex stabilization in the decoding centre^13^. It is plausible that during translocation, uS12 transmits SSU body back-rotation by pulling domain III of eEF2 that in turn retransmits it to switch II in the G-domain, stimulating GTP hydrolysis (Extended Data Fig. 4c, d). We found additional stabilization of domain IV by the N terminus of the eukaryote-specific protein eS30, which also interacts extensively with conserved decoding protein uS12 (Extended Data Fig. 4c). It can be assumed that eS30 co-evolved with eEF2, whose domain IV has 65 additional amino acids compared with its bacterial counterpart EF-G, to provide supplementary stabilization as well as enhancing propagation of conformational changes at the decoding site^13,14^. Other interactions between eEF2, tRNAs and the 80S ribosome are presented in Extended Data Figs. 5, 6.

## Diphthamide

**Fig. 2:**
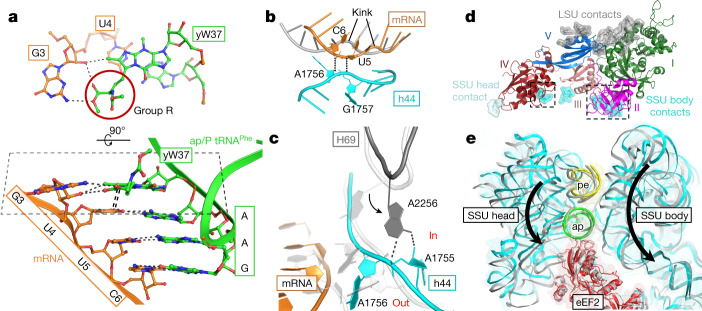
Stabilization of mRNA–tRNA by wybutosine modification of tRNA^Phe^, rearrangements of the decoding centre and depiction of a pawl function for eEF2 during translocation. **a**, Wybutosine cross-strand stacks on the first codon–anticodon base pair formed by mRNA codon UUC and tRNA^Phe^ at the ap/P state and stabilizes the last position of the adjacent upstream AUG codon (G3). An alternative view (bottom) of the same pattern clearly demonstrates how wybutosine enhances stabilization of mRNA to prevent a frame shift during translocation. **b**, Interactions between the backbones of translocating mRNA and decoding A1756 and A1757 of h44; mRNA in classical state is in grey. **c**, A shift of helix 69 (H69) of 25S rRNA is coupled to rearrangements of the decoding centre as compared to the bacterial classical state in white (PDB ID 4V6F). **d**, Contacts of eEF2 (coloured by domain) with LSU (grey), body (cyan) and head (pale cyan) of SSU; dashed squares indicate contacts that are disrupted at the late stage of translocation, allowing eEF2 to uncouple from the SSU body. **e**, Superposition of intermediate- and late (grey) (TI-POST-1, PDB ID 6GZ3)-translocation structures relative to LSU reveals eEF2 acting as a pawl anchoring and decoupling ap/P tRNA from the SSU head and body. The reverse direction of the SSU head and body rotation to a classical post-translocation state is indicated with arrows.

The current crystal structure of 80S ribosome translocation intermediate reveals direct involvement of diphthamide in stabilization of a codon–anticodon duplex in transition from the A- to P-site. eEF2 interacts with the mRNA codon exclusively via diphthamide, which protrudes into a cleft formed by mRNA, ap/P tRNA and rRNA (Fig. 1c, d). Assisted by His583 and Asp696 of eEF2, diphthamide contacts the codon–anticodon duplex minor groove. A similar pattern of interactions has been extensively described between the bacterial decoding centre mould and the codon–anticodon duplex in a classical unrotated state^13,14^ (Fig. 1d). The closest resemblance is observed at the second base pair (BP2, Fig. 1d), where diphthamide together with Asp696 mimics stabilization of decoding nucleotides G577 and A1755 in 18S rRNA (bacterial G530 and A1492).Fixation of the first codon–anticodon pair is divided between diphthamide with His583 and His694 contacting the anticodon ribose, and the wybutosine modification of ap/P tRNA^Phe^ of nucleotide 37 stacking on codon position +4 (BP1, Figs. 1d, 2a). The third codon–anticodon pair (BP3, Fig. 1d) is anchored by diphthamide only through its trimethylammonio moiety stabilizing the codon position. These findings are corroborated by numerous studies describing increased −1 frameshifting slippage when translation is performed with eEF2 lacking diphthamide^1,15^.

The mRNA backbone of the UUC codon paired to ap/P tRNA^Phe^ is located in close proximity to the sugar–phosphate backbone of decoding nucleotides A1755-A1756 and G1757, whose backbone is bulged out because of partial ‘flipped-in’ and ‘flipped-out’ positions of their nucleoside moieties (Fig. 2b). This close positioning of mRNA and A1756-G1757 backbones is realized by ribose–phosphate zipper interactions^16^. The diphthamide trimethylammonio moiety additionally stabilizes negatively charged backbones by interaction with the phosphate group between A1756 and G1757 of 18S rRNA (BP3, Fig. 1d).

**Extended Data Fig. 7:**
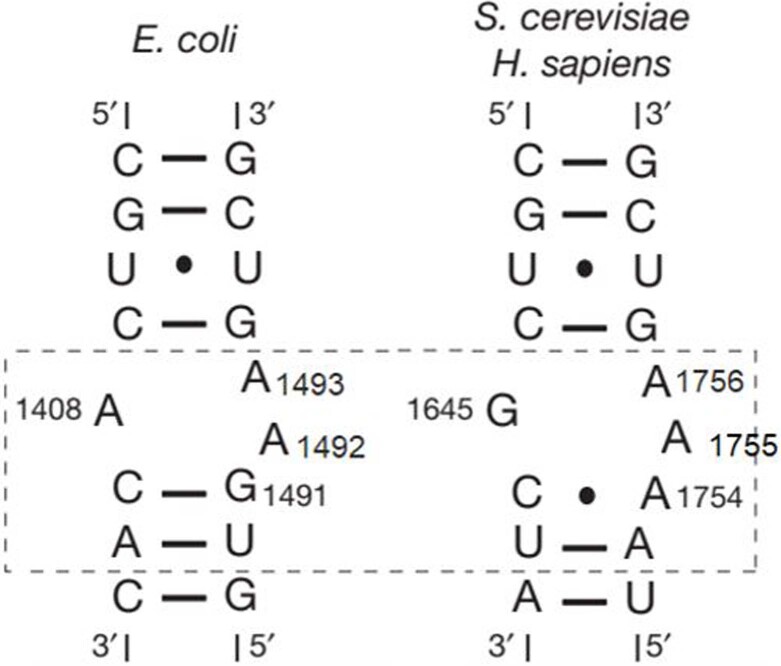
Comparison of secondary structures of the decoding loop in bacteria (left) and eukaryotes (right). In bacteria the internal loop of h44 of 16S rRNA consists of two nucleotides (A1492-A1493) on the 3’-side and has one nucleotide on the 5’-side (A1408). In contrast, eukaryotic 18S rRNA contains at least one additional nucleotide on each side is included (dashed box). Secondary structure diagrams of helix 44 from bacteria (16S rRNA, left) and from yeast and human (18S rRNA, right).

The difference in structure of helix 44 of SSU rRNA (h44), which forms a part of the decoding centre between bacteria and both eukaryotes and archaea might explain emergence of the diphthamide modification during evolution (Extended Data Fig. 7). The changes of decoding centre bulge, and flanking nucleotides could lead to increased flexibility of the eukaryotic decoding centre, which would require additional stabilization that was achieved by development of diphthamide modification of eEF2. In addition, the unique trimethylammonio moiety could have been evolutionally refined to reduce repulsion between juxtaposing negatively charged backbones of mRNA and the h44 decoding-centre loop at the early stages of translocation.

## Unlocking of the decoding centre

In the early-intermediate translocation state, when diphthamide takes over stabilization of the codon-anticodon duplex, decoding nucleotides rearrange to initiate resetting of the decoding centre. Nucleotide A1755 of 18S rRNA adopts a flipped-in position, whereas A1756 is in a more flipped-out and somewhat flexible conformation. There is a noticeable rearrangement of the pivotal intersubunit bridge B2a that is partially composed of A2256 of LSU helix 69 (H69) and decoding nucleotides of h44 (A1755–A1756) of SSU (Fig. 2c). In the present structure, adenosine 2256 of H69 protrudes towards sugar phosphate backbone between A1755–A1756, and contacts A1756 phosphate and A1755 ribose moieties. This connection allows H69 to transmit its movement directly to decoding adenines.

During selection of tRNA on the ribosome, the decoding centre serves as a mould imposing restraints on codon–anticodon nucleotides via defined interactions^13,14^ (Fig. 1d, right). To translocate mRNA by one codon, the decoding centre has to unlock from the bound codon–anticodon duplex. The present crystal structure with eEF2 suggests that destabilization of the decoding centre is initiated by anticlockwise rotation of SSU, which leads to rearrangement of the B2a bridge. The latterrearrangement induces displacement of H69 towards decoding h44, resulting in a new contact of A2256 of H69 with the decoding nucleotides A1755–A1756. Further rearrangements of the H69 tip lead to its synchronized movement with the decoding adenosines and triggers partial unlocking of the decoding centre from mRNA and tRNA (Fig. 2c). The trimethylammonio moiety of diphthamide contributes to these changes of A1755–A1756 and prevents decoding adenines from re-establishing their contacts with the codon–anticodon duplex (Fig. 1c, d).

Diphthamide-induced unlocking catalyses translocation by reducing the energy required for movement of the codon–anticodon pair. This function is supported by biochemical studies, which have demonstrated decreased protein synthesis rates in organisms lacking diphthamide^4,15^. Such an interpretation is also consistent with results of pre-steady-state kinetics studies on the bacterial translocation system^17^ and may explain the inhibitory effects of the antibiotics paromomycin and viomycin that interfere with the resetting of the decoding nucleotides by binding to the decoding centre region of h44 and reduce the rate of translocation by about 160- and more than 10,000-fold, respectively^18–20^.

## Wybutosine

In the current structure, we observe clear density for the tRNA^Phe^ hyper-modification wybutosine in position 37 in an authentic binding state on the ribosome. Wybutosine consists of wyosine base with 4-methoxy-3-[(methoxycarbonyl)amino]−4-oxobutyl group (group R; Fig. 2a, Extended Data Fig. 2c). Wyosine cross-strand stacks with the first codon nucleoside paired to ap/P tRNA. Group R stretches towards the third nucleotide (G3) of the codon coupled to the anticodon of pe/E tRNA and forms two hydrogen bonds between its methylcarboxyl portion and G3, hence, upgrading the triplet codon–anticodon interaction with ap/P tRNA to a quadruplet interaction (Fig. 2a). To our knowledge, this is the first time a tRNA modification has been seen to directly influence two adjoint codon–anticodon pairs by interactions with mRNA. Interactions between group R of wybutosine and the third position of the adjacent codon coupled to pe/E tRNA are possible because of a closer distance between ap/P tRNA and pe/E tRNA (Extended Data Fig. 2a). This situation is not achievable with tRNAs in classical A/A and P/P states and during the early stage of translocation where both tRNAs are positioned more than 10 Å apart, indicating that the main stabilization role of wybutosine modification is strongly manifested in the intermediate translocation states. This also implies that the (−1) ribosomal frameshifting events with hypomodified wybutosine tRNA are likely to occur during intermediate and late steps of translocation. These findings substantiate previous studies explaining why lack or alteration of wybutosine derivatives intRNA^Phe^ increases the (−1) programmed ribosomal frameshifting frequency and is associated with poor survival in patients with cancer^2,21^, and also demonstrates how the presence of wybutosine influences the efficiency of viral ribosomal frameshifting^22^.

## Conclusion

**Fig. 3:**
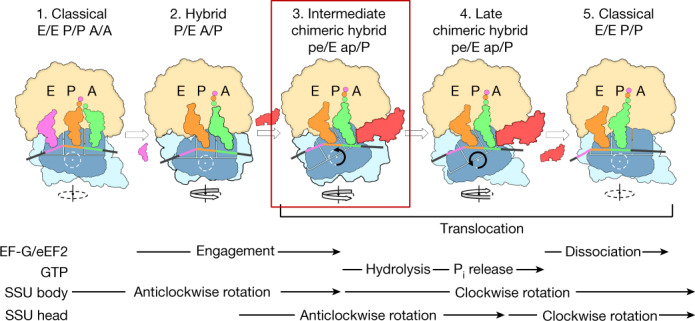
Integrating kinetic and structural studies of translocation. Top, translocation scheme based on the crystal structure of the intermediate translocation complex reported here (in frame) and on cryo-EM structures of late translocation (PDB ID: 6GZ3 and 6GZ5), as well as hybrid and classical post-translocation states (PDB ID: 3J77 and 3J78). A proposed sequence of events based on kinetic studies^28^ is shown at the bottom. Steps 1 and 2, thermally driven intersubunit rotations lead to tRNAs adopting hybrid A/P and P/E states and eEF2–GTP binding to the 80S ribosome. Steps 2 and 3, concomitant changes of LSU H69 composing intersubunit bridge B2a and the decoding centre, and insertion of the eEF2 diphthamide to the SSU A-site induce unlocking of the decoding centre. The released codon–anticodon duplex becomes stabilized by direct interactions with diphthamide. Detachment of tRNA ASLs from the SSU body and further insertion of the eEF2 domain IV into the A-site cause initial anticlockwise rotation of the head and movement of the second tRNA from the SSU P-site towards the E-site where it binds to L1 stalk. Steps 3 and 4, eEF2 remains anchored to LSU via domains I and V but is released from SSU where domain IV uncouples tRNA–mRNA from rearrangements of the SSU body and head. What is perceived as an additional large swivelling of the head is actually a result of the body back-rotation while the head remains fixed to tRNAs. Step 4 and 5, this body rotation increases the strain in the SSU neck and leads to uncoupling of the head from tRNAs. Formation of contacts between rRNA of the head and domain IV of eEF2 restrain the head position. The last steps of translocation are achieved when the head, owing to the increasing strain on the neck, snaps back to a non-rotated state and tRNA–mRNA binds to the SSU P-site environment.

The unique state of the translocating eukaryotic ribosome described here demonstrates eEF2 functioning as a pawl^3^ during translocation (Figs. 2d, 3, Extended Data Fig. 4a, b). Comparison of the early translocation-intermediate complex described here with the late translocation-intermediate TI-POST-1^6^ shows that eEF2 is sturdily anchored on LSU via domains I and V (Fig. 2e, Supplementary Video) with tRNAs retaining very similar positions in both states of the 80S ribosome, while SSU head and body move around them. The comparison suggests that domain IV of eEF2 uncouples tRNA–mRNA from the SSU body and head allowing these domains to return to their pretranslocation positions without pulling tRNA and mRNA back with them. Interestingly, an increase in the head swivel from around 13.5° (in the early intermediate) to about 18° (in TI-POST-1) is a result of the body back-rotation that thehead cannot follow because it remains attached to tRNAs (Fig. 2e). This probably creates a strain in the SSU neck region that eventually forces the head to unbind from tRNAs and to rotate back (Supplementary Video). Thus, the last steps of translocation are achieved by back retracement of the head to a non-rotated state and rebinding of tRNA–mRNA (one codon further down) to the SSU P-site environment.

The translocation process is enabled by Brownian intersubunit rotations of the ribosome^23,24^. The GTP-bound state of eEF2 on the ribosome stays in rigid conformation, as it is observed in current structure of early-translocation intermediate as well as in late-translocation intermediates^6^ (Fig. 2d, e), thus serving as a pawl that ensures directionality of translocation process. The described model supports findings showing that the energy stored in the eEF2•GTP state is sufficient to promote translocation^25^ and suggests that hydrolysis of GTP does not occur until the late steps of the process. It has been shown previously that domain IV of eEF2•GTP state can reach a codon–anticodon duplex of the P-site tRNA of the non-rotated ribosome^6^. However, experiments using fluorescence resonance energy transfer and other techniques^6,26^ have reported that translocation induced by eEF2 with a non-hydrolyzable analogue of GTP is prone to reversion, demonstrating a critical role of GTP hydrolysis in promoting unidirectionality of translocation. GTP hydrolysis and inorganic phosphate release are most likely to occur during the late steps of translocation during SSU body back-ratcheting, when hydrolysis of GTP is stimulated by the movement of uS12–eEF2 domain III (Extended Data Fig. 4c, d). Conformational changes of eEF2 induced by GTP hydrolysis enable unbinding from the mRNA–tRNA module in a manner that prevents pulling the codon–anticodon duplex back to the A-site. Similarly, the bacterial homologue of eEF2 undergoes a large rotation in domain III before its dissociation from the ribosome^27^.

## Methods

### 80S ribosome purification

Purification of the 80S ribosomes from the JD1370-∆Stm1 yeast strain^7^ was carried out according to the previously described protocol^29^, with some modifications. The crude ribosomes obtained by precipitation with 8.5% PEG 20,000 were re-suspended in buffer M (30 mM Hepes-KOH, 10 mM MgCl_2_, 50 mM KCl, 8.5% mannitol, 0.5 mM EDTA-KOH, 2 mM DTT, pH 7.55) and MgCl_2_ and KCl concentrations were slowly adjusted to 10 and 500 mM (10/500), respectively. The ribosomal suspension was then incubated on ice for 35 min with mild vortexing. The ribosomes were applied on the 6% sucrose cushion, which was prepared in the same dissociation conditions (10/500) and layered over the 10–30% sucrose gradient as in ref. ^7^ with 5 mM spermidine (5 mg of ribosomes per SW28 tube). After the overnight centrifugation selected fractions of the 80S ribosomes were collected and the ribosomes were precipitated by PEG 20,000.

### Purification of native eEF2

The isolation procedure of native eEF2 was mainly based on the previously described protocol^30^, with changes in several steps. First, we used a fresh culture of yeast strain JD1370-∆Stm1 grown to an *A*_600_ of 5–6 and cells were lysed in a microfluidizer. Second, instead of S-Sepharose, source-Q and uno-Q ion-exchange columns we used SP-Sepharose, Q-Sepharose and introduced a gel filtration with Sephadex-200 as the final purification step. The final sample was stored in pH 7.5 buffer consisting of 20 mM Tris-HCl, 5 mM MgCl_2_, 100 mM KCl, 10% glycerol and 1 mM DTT.

### Purification and aminoacylation of tRNAs

*S. cerevisiae* tRNA^Phe^ (‘chemical block’) was aminoacylated according to the protocol as described^31^ with minor modifications. After three rounds of phenol–chloroform–isoamyl alcohol extraction *S. cerevisiae* Phe-tRNA^Phe^ was purified on a column DeltaPack, C4 300A, 5mkm, 3.9 × 150 mm HPLC column (Waters) using a ethanol gradient as described^32^. The final sample was stored in 20 mM NH_4_CH_3_CO_2_ pH 5.0 at −80 °C. *Escherichia coli* tRNA^fmet^ was prepared and then aminoacylated and formylated according to^33^. After phenol extraction, fMet-tRNA^fmet^ was purified by hydrophobic chromatography using TSK-gel Phenyl-5PW column, and the final sample was stored in 20 mM NH_4_CH_3_CO_2_ pH 5.0 at −80 °C.

### 80S ribosome translocation complex formation

For reconstitution of translocation complex *S. cerevisiae* 80S ribosomes (2.2 μM) and 5′-AAUGUUCAA-3′ mRNA (Dharmacon) (2.9 μM) were incubated at 30 °C for 10 min in 6 mM Mg(CH_3_COO)_2_, 50 mM KOAc, 10 mM NH_4_Cl and 1.25mM DTT, 10 mM Hepes-KOH (pH 7.5). The fMet-tRNA^fMet^ (2.9 μM) was added and the complex further incubated for 7 min at 30 °C with following addition of Phe-tRNA^Phe^ (6.5 μM). The complex was incubated for additional 7 min at 30 °C. Separately, *S. cerevisiae* eEF2 (8.7 μM) was incubated with GDPCP (Jena Bioscience) (0.25 mM) for 10 min at room temperature and mixed with the ribosome complex for a final incubation at 30 °C for 10 min. The detergent Deoxy Big CHAP (CalBioChem) (2.4 mM) was added and after 5 min at room temperature the complex was incubated at 4 °C for 5 min.

### Crystallization and crystal treatment

The 80S ribosome translocation complex was crystallized at 4 °C by vapour diffusion in the MRC-48 siting drop plates (Hampton Research) by mixing 3 µl of the complex with 3 µl of the reservoir solution (100 mM bis-Tris-HCl, pH 7.0, 300 mM NH_4_SCN, 100 mM KCl, 8.25% – 9.5% PEG 20K, 1 mM Mg(CH_3_COO)_2_, 2% glycerol, 1% sucrose, 5 mM putrescine). Crystals appeared after 3 days and grew to their full size in 13 days.

The post-crystallization treatment was carried out via dehydrationby replacing reservoir solution with saturated MgCl_2_ salt and incubating for 18 h. Treatment solution (3.3% PEG 20K, 6% PEG 10K, 115 mM bis-Tris-HCl, pH 5.4, 18 mM putrescine, 21 mM Mg(CH_3_COO)_2_, 9% glycerol, 0.75% sucrose, 1.8 mM deoxy big CHAP, 2.3 mM DTT) was added to the crystallization drop before dehydration. The crystals were collected and flash-frozen in liquid nitrogen.

### Data collection and structure refinement

Data collection was carried out at 100 K at beamline PX1 - X06SA at the Swiss Light Source synchrotron at 1.0 Å wavelength using DA+ data acquisition and analysis software^34^. Data were integrated and scaled with the XDS program^35^. The search model was generated from the previously published structure of the yeast 80S ribosome^29^ (PDB ID 4V88). The initial molecular replacement solution was refined in PHENIX by rigid-body refinement with the 40S and 60S subunits treated as rigid bodies. After initial refinement, density corresponding to the mRNA, tRNAs, eEF2 as well as ribosome rearrangements became clearly visible in the difference electron density map. The crystal structure of eEF2 (PDB ID 1N0U) was docked into the density and manually adjusted before refinement. Refinement was carried out in alternating cycles of automated refinements using PHENIX with manual refinement and model building in COOT resulting in a model with Ramachandran favoured, allowed and outliers of 83.4%, 14.4% and 1.1% respectively. A summary of refinement and data collectionstatistics is displayed in Extended Data Table 1. All figures were generated using PyMOL.

### Reporting summary

Further information on research design is available in the Nature Research Reporting Summary linked to this paper.

## Online content

Any methods, additional references, Nature Research reporting summaries, source data, extended data, supplementary information, acknowledgements, peer review information; details of author contributions and competing interests; and statements of data and code availability are available at 10.1038/s41586-021-04131-9.

### Supplementary information

Reporting Summary

Supplementary Video 1 Translocation of tRNAs on the 40S ribosome subunit and codon–anticodon stabilization by diphthamide and wybutosine. Intersubunit movement in the 80S ribosome and internal conformational changes of the 40S ribosomal subunit during eEF2 catalysed translocation of tRNAs. The movie is based on the presented crystal structure of early translocation-intermediate complex (PDB ID 7OSM), as well as cryo-EM structures of late translocation (PDB ID 6GZ3, 6GZ5).

## Figures and Tables

**Extended Data Table 1:**
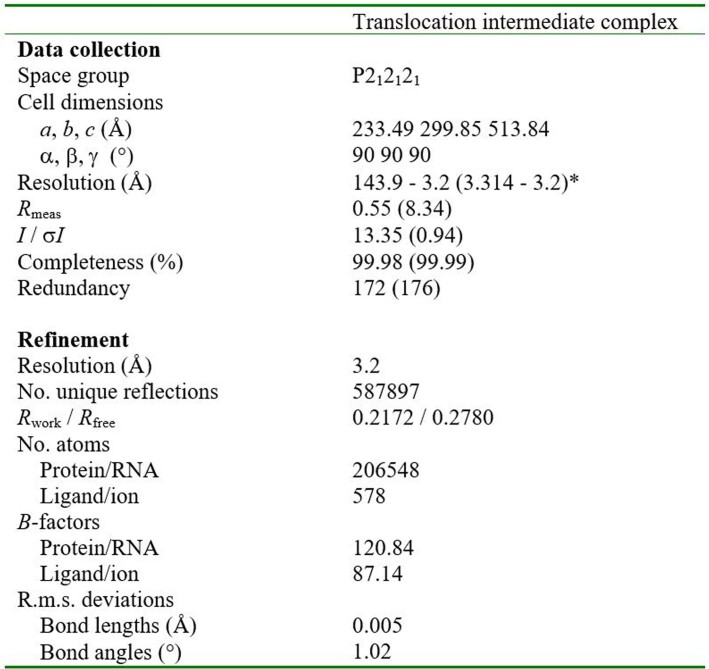
Data collection and refinement statistics

A total of 8 crystals were used for this dataset.

*Values in parentheses are for highest-resolution shell.

## Data Availability

Coordinates and structure factors have been deposited with the Protein Data Bank under accession code 7OSM. Previously published models that were used for analysis and comparison are also available in the Protein Data Bank with accession codes 4V88, 4V6F, 1N0U, 3J77, 3J78, 4V6F, 6GZ3 and 6GZ5.
