# Supplementary material for: Accuracy mechanism of eukaryotic ribosome translocation
Source: Nature. 2021 Dec 1;600(7889):543–6. doi: 10.1038/s41586-021-04131-9 (PMC8674143; doi:10.1038/s41586-021-04131-9)
Supplement: Supplementary file 1 — Reporting Summary [file 41586_2021_4131_MOESM1_ESM.pdf]

nature portfolio

Corresponding author(s): Marat Yusupov, Gulnara Yusupova

Last updated by author(s): Oct 4, 2021

Reporting Summary

Nature Portfolio wishes to improve the reproducibility of the work that we publish. This form provides structure for consistency and transparency in reporting. For further information on Nature Portfolio policies, see our Editorial Policies and the Editorial Policy Checklist.

Statistics

For all statistical analyses, confirm that the following items are present in the figure legend, table legend, main text, or Methods section.

n/a	Confirmed	
☒	☐	The exact sample size (*n*) for each experimental group/condition, given as a discrete number and unit of measurement
☒	☐	A statement on whether measurements were taken from distinct samples or whether the same sample was measured repeatedly
☒	☐	The statistical test(s) used AND whether they are one- or two-sided<br>*Only common tests should be described solely by name; describe more complex techniques in the Methods section.*
☒	☐	A description of all covariates tested
☒	☐	A description of any assumptions or corrections, such as tests of normality and adjustment for multiple comparisons
☒	☐	A full description of the statistical parameters including central tendency (e.g. means) or other basic estimates (e.g. regression coefficient) AND variation (e.g. standard deviation) or associated estimates of uncertainty (e.g. confidence intervals)
☒	☐	For null hypothesis testing, the test statistic (e.g. *F*, *t*, *r*) with confidence intervals, effect sizes, degrees of freedom and *P* value noted<br>*Give P values as exact values whenever suitable.*
☒	☐	For Bayesian analysis, information on the choice of priors and Markov chain Monte Carlo settings
☒	☐	For hierarchical and complex designs, identification of the appropriate level for tests and full reporting of outcomes
☒	☐	Estimates of effect sizes (e.g. Cohen's *d*, Pearson's *r*), indicating how they were calculated

Our web collection on statistics for biologists contains articles on many of the points above.

Software and code

Policy information about availability of computer code

Data collection	Crystallographic data were collected at PX1 - X06SA beamline of The Swiss Light Source (SLS) synchrotron at the Paul Scherrer Institut using DA+ data acquisition and analysis software
Data analysis	XDS build 20190315<br>PHENIX 1.14-3260<br>COOT 0.8.9<br>PyMol 2.2.0

For manuscripts utilizing custom algorithms or software that are central to the research but not yet described in published literature, software must be made available to editors and reviewers. We strongly encourage code deposition in a community repository (e.g. GitHub). See the Nature Portfolio guidelines for submitting code & software for further information.

Data

Policy information about availability of data

All manuscripts must include a data availability statement. This statement should provide the following information, where applicable:

- Accession codes, unique identifiers, or web links for publicly available datasets
- A description of any restrictions on data availability
- For clinical datasets or third party data, please ensure that the statement adheres to our policy

The atomic coordinates and structure factors for the determined crystal structure have been deposited in the Protein Data Bank with the accession code 7OSM (translocation intermediate complex). Previously published models that were used for analysis and comparison are also available from the Protein Data Bank with accession codes 4V88, 4V6F, 1N0U, 3J77, 3J78, 4V6F, 6GZ3, 6GZ5.

Field-specific reporting

Please select the one below that is the best fit for your research. If you are not sure, read the appropriate sections before making your selection.

☒ Life sciences ☐ Behavioural & social sciences ☐ Ecological, evolutionary & environmental sciences

For a reference copy of the document with all sections, see nature.com/documents/nr-reporting-summary-flat.pdf

Life sciences study design

All studies must disclose on these points even when the disclosure is negative.

Sample size	The data set was complete and with a high redundancy as can be seen from the data statistics in Table 1 in accordance with generally accepted crystallographic methods.
Data exclusions	No data were excluded from analysis. During data processing heterogeneous crystals which did not scale correctly with the main dataset were removed from further consideration.
Replication	All biochemical and crystallization procedures were successfully replicated more than 5 times in independent experiments.
Randomization	No randomization was required for the reported experiments, as all variables could be controlled.
Blinding	Blinding is not applicable to crystallographic experiments. When needed during analysis of the models we compared our complex structure with previously published native structures.

Reporting for specific materials, systems and methods

We require information from authors about some types of materials, experimental systems and methods used in many studies. Here, indicate whether each material, system or method listed is relevant to your study. If you are not sure if a list item applies to your research, read the appropriate section before selecting a response.

Materials & experimental systems

n/a	Involved in the study	
☒	☐	Antibodies
☒	☐	Eukaryotic cell lines
☒	☐	Palaeontology and archaeology
☒	☐	Animals and other organisms
☒	☐	Human research participants
☒	☐	Clinical data
☒	☐	Dual use research of concern

Methods

n/a	Involved in the study	
☒	☐	ChIP-seq
☒	☐	Flow cytometry
☒	☐	MRI-based neuroimaging
